# Identification, Characterization, and Immobilization of an Organic Solvent-Stable Alkaline Hydrolase (PA27) from *Pseudomonas aeruginosa* MH38

**DOI:** 10.3390/molecules190914396

**Published:** 2014-09-12

**Authors:** Eunjin Jang, Bum Han Ryu, Thomas Doohun Kim

**Affiliations:** Department of Applied Chemistry and Biological Engineering, College of Engineering, Ajou University, Suwon 443-749, Korea; E-Mails: thomas70kim@gmail.com (E.J.); clara-y@hanmail.net (B.H.R.)

**Keywords:** PA27, hydrolase, immobilization, stability, electron microscopy

## Abstract

An organic solvent-stable alkaline hydrolase (PA27) from *Pseudomonas aeruginosa* MH38 was expressed, characterized, and immobilized for biotechnological applications. Recombinant PA27 was expressed in *Escherichia coli* as a 27 kDa soluble protein and was purified by standard procedures. PA27 was found to be stable at pH 8–11 and below 50 °C. It maintained more than 80% of its activity under alkaline conditions (pH 8.0–11.0). Furthermore, PA27 exhibited remarkable stability in benzene and *n*-hexane at concentrations of 30% and 50%. Based on these properties, immobilization of PA27 for biotechnological applications was explored. Scanning electron microscopy revealed a very smooth spherical structure with numerous large pores. Interestingly, immobilized PA27 displayed improved thermal/chemical stabilities and high reusability. Specifically, immobilized PA27 has improved thermal stability, maintaining over 90% of initial activity after 1 h of incubation at 80 °C, whereas free PA27 had only 35% residual activity. Furthermore, immobilized PA27 showed higher residual activity than the free enzyme biocatalysts against detergents, urea, and phenol. Immobilized PA27 could be recycled 20 times with retention of ~60% of its initial activity. Furthermore, macroscopic hydrogel formation of PA27 was also investigated. These characteristics make PA27 a great candidate for an industrial biocatalyst with potential applications.

## 1. Introduction

Lipolytic enzymes from microorganisms are one of the most important biocatalysts for a wide range of industrial applications in the textile, cosmetic, paper, food, and pharmaceutical industries [1,2,3]. These enzymes are gradually replacing inorganic chemical catalysts in industrial processes due to the high yields of products, strong selectivity of products, and less environmental risks they offer. Specifically, enzymes that can retain their activity in the presence of organic solvents are highly attractive and important, as they can be used in many chemical reactions [4,5].

After an early study on an organic solvent stable lipase from *Pseudomonas* *aeroginosa* LST-03 [6], significant efforts have been done to identify novel organic solvent-stable enzymes from *Pseudomonas aeruginosa*, *Stenotrophomonas maltophilia*, and *Bacillus sphaericus*. To date, several organic solvent stable lipases were identified and characterized, including LipS [7], SML [8], LipYY31 [9], LC2-8 [10], PseA [11], and 205y lipase [12]. However, the number of organic solvent stable lipases is still limited, and the search for novel enzymes to satisfy the needs of practical applications is of high importance. In the present study, a novel organic solvent-stable alkaline hydrolase (PA27) from *Pseudomonas aeruginosa* MH38 was identified, characterized, and immobilized. Furthermore, crosslinked enzyme aggregrates and hydrogel formation of this enzyme was also discussed.

## 2. Results and Discussion

### 2.1. Biochemical Analysis of PA27

In the genome sequence of *P. aeruginosa* MH38 (GenBank: CBTQ010000036), an open reading frame (ORF; locus tag: P38_1135) of 747 bp, which encoded a protein of 248 amino acids with pI of 5.3, was identified (Locus: CDH69439, Uniprot code: W0WBF0). PA27 showed high sequence similarity to a carboxylesterase PA3859 (85% sequence identity, PDB I.D.: 3CN7) from *Pseudomonas aeruginosa* [13] and a carboxylestease from *Pseudomonas fluorescens* (65% sequence identity, PDB I.D.: 1AUO) [14].

**Figure 1:**
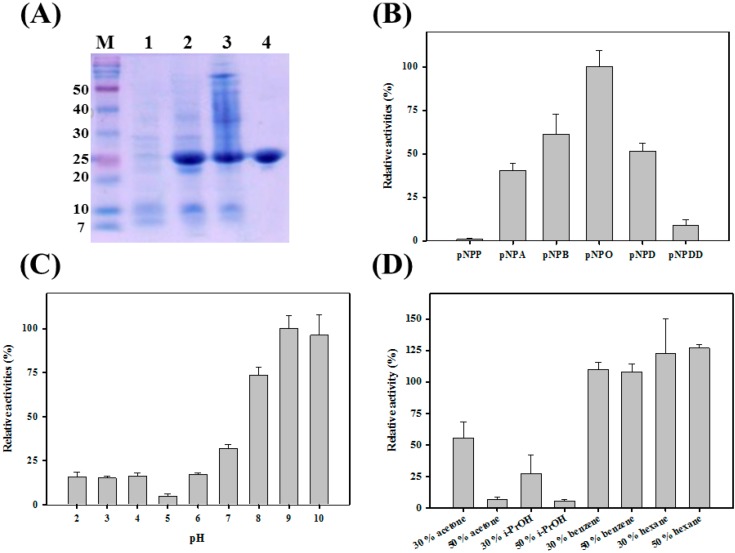
(**A**) SDS-PAGE analysis of PA27. Lane M, marker, lane 1 & 2; crude *Escherichia coli* extracts before and after induction; lane 3, supernatants of *E. coli* extracts; lane 4, PA27 after dialysis; (**B**) Substrate specificity of PA27 was investigated towards different *p*-NP esters; (**C**) pH stability of PA27; (**D**) Organic solvent-stable properties of PA27.

To study the catalytic properties of PA27, recombinant PA27 was purified to near homogeneity using Ni-NTA affinity chromatography. As indicated by the single band in the SDS-PAGE gel, the purified protein was homogeneous (Figure 1A). The hydrolyzing activity of PA toward *p*-NP esters of different acyl chain lengths was analyzed. As shown in Figure 1B, PA27 displayed a strong preference toward *p*-nitrophenyl octanoate (*p*-NPO), although it could not hydrolyze *p*-nitrophenyl phosphate (*p*-NP) and very little activity was observed for *p*-nitrophenyl dodecanoate (*p*-NPDD).

In addition, the activity profile of PA27 at different pH values was investigated in the range of 2.0–10.0 (Figure 1C). PA27 displayed its maximal activity at pH 9.0 and ~95% and ~70% of its maximal activity was observed at pH 10.0 and 8.0, respectively. However, only ~20% of the maximal activity was retained at pH 6.0. Activity above pH 10.0 could not be properly measured because of the autohydrolysis of the substrates.

As shown in Figure 1D, PA27 showed ~58% of its initial activity in the presence of 30% (v/v) acetone, and it retained only ~25% activity in the presence of 30% (v/v) isopropanol (*i*-PrOH). However, PA27 was almost inactive and retained ~3% activity in the presence of 50% acetone or 50% *i*-PrOH. Interestingly, as shown in Figure 1D, enzyme activity of PA27 was enhanced in benzene and hexane. The activity of PA27 was as high as ~110% and ~125% of its original activity in the presence of 30% benzene and 50% hexane, respectively. Similar behaviors were also observed in LC2-8 or PseA [10,11,15].

**Figure 2:**
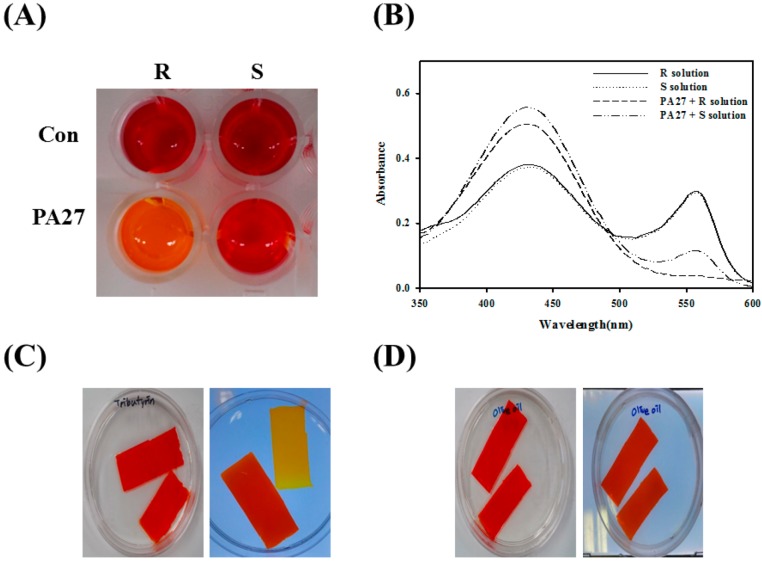
(**A**) pH shift assay was conducted in the presence of (*R*)- or (*S*)-methyl (*R*)-3-hydroxy-2-methylpropanoate; (**B**) The absorbance spectrum of each solution after 1 h was measured; (**C**,**D**) Activity staining of PA27 in non-denaturing gel towards glyceryl tributyrate (C) and olive oil (D).

For the enantioselectivity analysis of PA27, a pH shift assay was used with (*R*)- and (*S*)-methyl (*R*)-3-hydroxy-2-methylpropanoate (Figure 2A). Lipolytic activity was detected based on the color change of the phenol red indicator due to acid release. After incubation with PA27, the color of the reaction mixture turned yellow in (*R*)- and (*S*)-enantiomer-containing solutions, which was also confirmed by absorbance spectra readings (Figure 2B). The results show that PA27 prefers to hydrolyze the (*R*)-enantiomer when compared to its (*S*)-enantiomer. The catalytic properties of PA27 were investigated by activity-based native gel staining using glyceryl tributyrate and olive oil. A yellowish color was detected only in solutions containing glyceryl tributyrate, which indicated the formation of hydrolysis products. However, PA27 could not effectively hydrolyze olive oil, because no color changes were developed under these conditions (Figure 2C,D).

An interesting feature of PA27 is its ability to form hydrogels, which was reminiscent of hen egg white lysozyme [16]. In addition, a variable degree of aggregation was also reported in an extracellular lipase from *Pseudomonas aeruginosa* EF2 [17]. 

**Figure 3:**
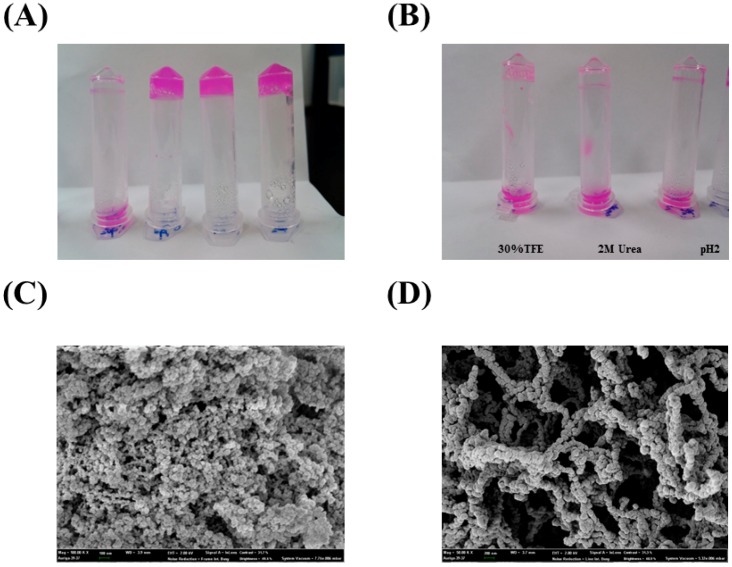
(**A**) Hydrogel formation was investigated under several conditions (20 mM DTT, buffer only, 1 M NaCl, and 1 mM Cu^2+^, (from right to left); (**B**) Inhibition of hydrogel formation by 30% TFE, 2 M urea, and 50 mM glycine-HCl (pH 2.0); (**C**,**D**) SEM images of the PA27 hydrogel. Representative images at 50 kX (C) and 100 kX (D) are shown.

Hydrogels were prepared by dissolving PA in buffer solution (20 mM Tris-HCl, Ph 8.0) with heating at 80 °C for 10 min and then cooling at 25 °C. No gel was observed for the equivalent sample with 20 mM DTT. Although the hydrogel formation of PA in the presence of 1 M NaCl and 1 mM Cu^2+^ was observed, 20 mM DTT, 2 M urea, and 20 mM glycine·HCl (pH 2.0) strongly inhibited the formation of PA hydrogels (Figure 3A,B). In the SEM images, the surface of the PA27 hydrogels resembled three-dimensional networks of globular shapes (Figure 3C,D).

### 2.2. Immobilization of PA27

**Figure 4:**
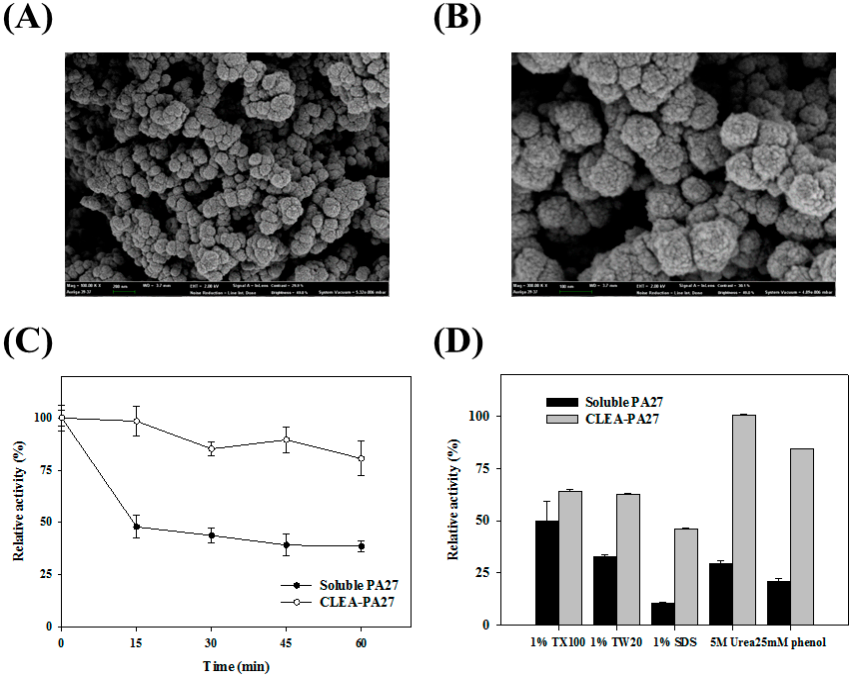
(**A**,**B**) Scanning electron microscopy (SEM) images of immobilized PA27. Images at 50 kX (A) and 100 kX; (**C**) Thermostability of immobilized PA27 (○) and soluble PA27 (●) at 80 °C; (**D**) The chemical stability of immobilized PA27 and soluble PA27 was compared.

For immobilization, crosslinked enzyme aggregates of PA27 (CLEA-PA27) were formed by precipitating enzymes in the presence of 80% AMS with glutaraldehyde crosslinking [18]. The SEM images of the immobilized PA27 showed mainly globular structures with high packing density (Figure 4A,B). For thermostability analysis, soluble PA27 as well as immobilized PA27 were incubated for 1 h at 70 °C and the enzyme activity was measured at various time intervals. After 1 h of incubation, free PA27 showed ~30% of its initial activity, while immobilized PA27 retained most of its initial activity (Figure 4C). 

The effects of several chemicals on the activity of immobilized PA27 and soluble PA27 were compared (Figure 4D). As expected, immobilized PA27 showed more resistance against these chemicals (*i.e.*, Triton X-100, Tween 20, SDS, urea, and phenol) than soluble PA27. As shown in Figure 4D, immobilized PA27, compared to soluble PA27, was appreciably stable in the presence of Triton X-100 and Tween 20. SDS was found to be a strong inhibitor of PA 27. Specifically, 1.0% (v/v) SDS almost completely deactivated soluble PA27, even though immobilized PA27 retained as high as ~40% of its initial activity. Moreover, in the presence of 5 M urea, soluble PA27 showed only ~30% of its activity compared to ~105% of the immobilized PA27. Therefore, the immobilization of PA27 could effectively protect the enzymes from inactivation for a variety of industrial applications. For reusability, immobilized PA27 was reused repeatedly for 20 sequential cycles, retaining ~60% of its initial activity (Table 1).

**Table 1:** Reusability of immobilized PA27 after 20 cycles.

Cycle Number	Relative Activity
10	70.2 ± 10.8
15	66.9 ± 8.0
20	60.7 ± 7.1

## 3. Experimental Section

### 3.1. Bacterial Strains, Reagents, and Chemicals

*Escherichia coli* strains (DH5α and BL21 (DE3)) were obtained from Stratagene (La Jolla, CA, USA) and Merck Millipore (Darmstadt, Germany), respectively. The Ni-NTA affinity column was purchased from GE Healthcare Korea (Seoul, Korea). Molecular biology enzymes and chemical compounds, including methyl (*R*)-3-hydroxy-2-methylpropanoate (99%) and methyl (*S*)-3-hydroxy-2-methylpropanoate (99%) were obtained from New England Biolabs (Ipswich, MA, USA) and Sigma Aldrich Korea (Yongin, Korea), respectively.

### 3.2. Cloning and Purification of PA27

PA27 gene was amplified from the chromosomal DNA of *P. aeruginosa* MH 38 by polymerase chain reaction (PCR). Restriction enzyme sites were added (5′-*Nde*I and 3′-*Xho*I) to allow subcloning into the pET-21a expression vector. After DNA sequencing, the resulting plasmid (pET-PA27) was transformed to express PA27 in *E. coli* BL21 (DE3) cells. A single colony of *E. coli* BL21 (DE3) was inoculated into LB medium containing ampicillin (100 µg/mL) and incubated at 37 °C until OD_600_ reached ~0.6. Then, isopropyl β-d-thiogalactopyranoside (IPTG) was added to a final concentration of 0.5 mM, and bacteria were further cultured for 4 h. The bacterial cells were harvested and sonicated in cell lysis buffer (20 mM Tris-HCl (pH 8.0), 100 mM sodium chloride, and 20 mM imidazole). After centrifugation at 6000 rpm for 20 min at 4 °C, the supernatants were loaded onto a Ni-NTA column followed by extensive washing with cell lysis buffer. PA27 were then eluted with column buffer (20 mM Tris-HCl (pH 8.0), 100 mM sodium chloride, and 100 mM imidazole), and desalted with storage buffer (20 mM Tris-HCl (pH 8.0)) using a PD-10 column. The purity of recombinant PA27 was verified by sodium dodecyl sulfate-polyacrylamide gel electrophoresis (SDS-PAGE). The final protein was stored at −20 °C without further modifications.

### 3.3. Enzymatic Assays

Hydrolysis activity was determined by colorimetric assay using *p*-nitrophenyl ester substrates with varying alkyl chain lengths as previously described [19,20]. The activity of PA27 against *p*-nitrophenyl acetate (C_2_, *p*-NA), *p*-nitrophenyl butyrate (C_4_, *p*-NB), *p*-nitrophenyl octanoate (C_8_, *p*-NO), *p*-nitro-phenyl decanoate (C_10_, *p*-NDec), *p*-nitrophenyl dodecanoate (C_12_, *p*-NDo), and *p*-nitrophenyl phosphate (*p*-NP) was investigated. The reaction mixture contained 0.5 mL of 0.3 mM substrate solution in 50 mM sodium phosphate buffer, pH 7.5, and 0.1 µg of PA27 protein, which was incubated for 10 min, and the amount of *p*-nitrophenol was measured at 405 nm. The pH dependence of PA27 activity was determined after 1 h of incubation at 37 °C using different buffers: glycine·HCl (pH 2.5–3.5), citrate buffer (pH 3.0–6.0), phosphate buffer (pH 6.0–8.0), and glycine·NaOH (pH 9.0–11.0). For pH experiments, activity was measured with 10 mM *p*-nitrophenyl butyrate as the substrate in 20 mM Tris-HCl (pH 8.0). The effect of organic solvents (acetone, isopropanol (*i*-PrOH), benzene, and hexane) on the activity of PA27 was investigated by pre-incubating PA27 (0.1 µg) for 1 h in 20 mM Tris-HCl (pH 8.0) at 25 °C. For enantioselectivity analysis, PA27 was reacted with (*R*)- or (*S*)-solutions containing 2 g/L of phenol red and 300 mM (*R*)- or (*S*)-methyl (*R*)-3-hydroxy-2-methylpropanoate. Activity staining with non-denaturing native gel was conducted under non-reducing conditions (without SDS, 2-mercaptoethanol, and heat).

### 3.4. Immobilization of PA27

PA27 was immobilized by forming insoluble enzyme aggregates with ammonium sulfate (AMS) and crosslinking with glutaraldehyde [21,22]. Specifically, PA27 was precipitated with 80% (w/v) AMS at 4 °C. Then, 20 mM (final concentration 0.5 mM) glutaraldehyde was added and incubated for 12 h at 4 °C. The resulting suspension (immobilized PA27) was centrifuged at 12,000 rpm for 10 min, and washed five times with 20 mM Tris-HCl (pH 8.5) until enzyme activity was no longer detected in the supernatant. The activity of immobilized PA27 was determined by monitoring the hydrolysis of *p*-nitrophenyl octanoate (C_8_, *p*-NO). Precipitating soluble PA27 with AMS yielded ~75% residual activity compared with initial activity of PA27, which was also close to the reported data for other proteins [23,24]. Longer crosslinking time (up to 24 h) did not lead to any further changes in activity recovery. A scanning electron microscope (SUPRA 55VP, Carl Zeiss, Jena, Germany) was used to examine the surface morphology of the immobilized PA27. To determine the thermostability of the immobilized PA27 and soluble PA27, each form was incubated for 1 h at 80 °C. Aliquots of each sample were removed every 15 min and the residual activity was determined by monitoring the hydrolysis of *p*-nitrophenyl octanoate (C_8_, *p*-NO). The effects of chemicals (Triton X-100, Tween 20, SDS, urea, and phenol) on the activity of the immobilized PA27 and soluble PA27 were investigated after 1 h of incubation. The chemical stabilities were then determined by measuring the residual activity. The initial activity was defined as 100%. For reusability tests, the immobilized PA27 was recovered by centrifugation after each reaction and washed five times with 500 µL of 20 mM Tris-HCl (pH 8.5) until no significant enzyme activity was detected in the supernatant. Then, fresh enzyme substrate was added for another reaction. The immobilized PA27 was used for 20 cycles and these experiments were repeated three times.

### 3.5. Formation of PA27 Hydrogel

PA27 (1 mg/mL) was incubated with several compounds at 80 °C for 10 min. For staining, phenol red was added before heat treatment. The effect of several chemical compounds (*i.e.*, 30% TFE, 2 M urea, and 20 mM glycine-HCl, pH 2.0) on PA27 hydrogel formation was also investigated. Scanning electron microscopy was employed to investigate the surface morphology of the PA27 hydrogel.

## 4. Conclusions

This study reports on the biochemical characteristics of PA27, an organic solvent-stable alkaline hydrolase from *P. aeruginosa* MH38. The catalytic properties of PA27 such as high performance in alkaline pH, strong resistance to many organic solvents, and considerable compatibility with detergents, demonstrates its potential use for a wide range of biotechnological applications. Furthermore, immobilized PA27 was shown to be useful as a biocatalyst under extreme conditions, which renders it applicable as an effective catalyst in industrial bioreactors.
